# Icariin and its Derivative Icariside II Extend Healthspan via Insulin/IGF-1 Pathway in *C. elegans*

**DOI:** 10.1371/journal.pone.0028835

**Published:** 2011-12-21

**Authors:** Wai-Jiao Cai, Jian-Hua Huang, Su-Qin Zhang, Bin Wu, Pankaj Kapahi, Xin-Min Zhang, Zi-Yin Shen

**Affiliations:** 1 Institute of Traditional Chinese and Western Medicine, Huashan Hospital, Fudan University, Shanghai, China; 2 Buck Institute for Research on Aging, Novato, California, United States of America; 3 Clinical Medicine College of Hangzhou Normal University & The Second Hospital of Hangzhou affiliated to Hangzhou Normal University, Hangzhou, China; Roswell Park Cancer Institute, United States of America

## Abstract

Compounds that delay aging might also postpone age-related diseases and extend healthspan in humans. Icariin is a flavonol extracted from several plant species of the *Epimedium* family. The icariin and its metabolic derivatives have been shown to exert wide protective effects in age-related diseases. However, whether icariin and its derivatives have the potency of delaying aging remains unclear. Here, we report that icariin and its derivative icariside II extend *C. elegans* lifespan. Using HPLC, we found high level of icariside II in the animals treated with icariin, suggesting icariside II is the bioactive form *in vivo* of icariin. Icariside II also increased the thermo and oxidative stress tolerance, slowed locomotion decline in late adulthood and delayed the onset of paralysis mediated by polyQ and Aβ_1–42_ proteotoxicity. The lifespan extension effect of icariside II is dependent on the insulin/IGF-1 signaling (IIS) since the *daf-16(mu86)* and *daf-2(e1370)* failed to show any lifespan extension upon icariside II treatment. Consistently, icariside II treatment upregulates the expression of DAF-16 targets in the wild-type. Moreover, our data suggests that the heat shock transcription factor HSF-1 has a role in icariside II-dependent lifespan extension further implicating the IIS pathway. In conclusion, we demonstrate a novel natural compound, icariside II as the bioactive form of icariin, extends the healthspan via IIS pathway in *C. elegans*.

## Introduction

A major goal of current research on aging is to identify compounds that delay age-related diseases and extend healthspan in humans. *Herba epimedii* is a popular herbal tonic used in traditional Chinese medicine, with proven efficacy in treating several age-related diseases including osteoporosis, cardiovascular diseases, neurodegenerative diseases and sexual dysfunction [1], [2]. Icariin is the major pharmacologically active flavonol diglycoside of *Herba epimedii*. Various studies indicate the anti-oxidative effect of icariin on DNA damage, β-amyloid mediated neurotoxicity, and vein endothelial cell oxidative injury [1]. Meanwhile, icariin and its derivatives function as signaling modulators to exert beneficial effects in a multitude of age-dependent disease states, including bone loss, cancer, cardiovascular disease, and neurodegenerative disorders [3], [4], [5], [6]. However, whether icariin and its derivatives own the potency of slowing aging remain elusive. Our previous studies on *Epimedium* Flavones (EF), the raw extract of *Epimedium* which contains icarrin as a major constituent, show that EF delays aging in *Drosophila melanogaster* and *Caenorhabditis. elegans* [7], [8]. Interestingly, EF also resets the age-related metabolites (fatty acids, carnosine, ergothioneine and deoxycholic acid *et al*) to the juvenile level in rat plasma and urine [9], [10]. These findings further prompted us to investigate the anti-aging potential of icariin and its derivatives.

The experimental organism, *Caenorhabditis elegans* is a well-established aging model, sharing similar aspects of aging with mammals, such as sarcopenia and locomotion decline [11]. *C. elegans* has provided critical insight on conserved lifespan-regulated pathways, for example insulin/insulin-like growth factor signaling (IIS) [12], making *C. elegans* a model system to identify novel genetic links and compounds that can promote healthy aging in humans.

The highly conserved IIS pathway plays a key role in aging. It has been demonstrated in multiple species that inhibition of the IIS pathway extends lifespan [12]. In *C. elegans*, the *daf-2* gene encodes an insulin/IGF-1 receptor. Mutations in *daf-2* suppress the IIS which lead to the nuclear localization of FOXO/DAF-16 transcription factor. The activated FOXO/DAF-16 regulates a series of genes involved in lifespan control, stress tolerance and protein misfolding suppression [13], [14]. Nuclear localization of DAF-16 in the *daf-2* mutants requires the heat shock transcription factor (HSF)-1, which modulates the expression of heat shock proteins and protease responsible for the stress tolerance and protein folding [15]. Despite the well established role of a number of genes in the IIS pathway in modulating aging, pharmacological tools that inhibit IIS pathway to extend lifespan are not commonly available, which limits its translation to mammalian model systems.

In this study, we tested the anti-aging properties of icariin and its three derivatives, icariside I, icariside II and icaritin in *C. elegans*. We found that icariin and one of its derivatives, icariside II prolonged adult lifespan. Chemical profiles of icariin treated animals revealed that icariside II was the predominant bioactive form of icariin *in vivo*. Additionally, we found that icariside II treated animals have delayed age-associated phenotypes suggesting icariside II enhances the healthy aging significantly. Finally, our genetic analysis indicates that Icariside II may act through the IIS pathway to affect *C. elegans* lifespan.

## Results

### Icariin and its active metabolite icariside II extend *C. elegans* lifespan

**Figure 1:**
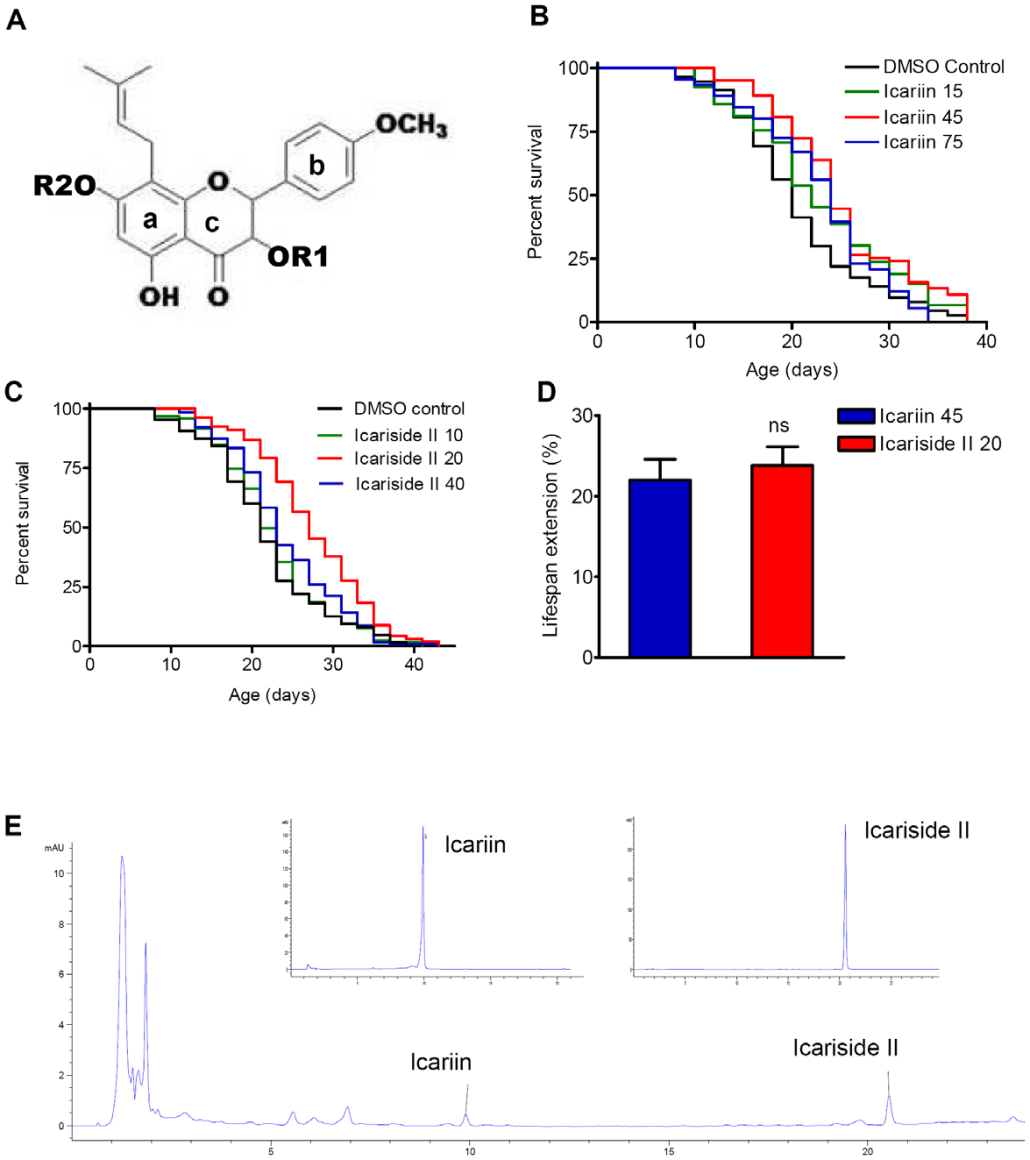
Icariin and its bioactive form icariside II extend lifespan in *C. elegans*. A. Chemical structures of icariin and its derivatives. The common structure is 8-prenylkaempferol. R1 and R2 are substituted by rhamnose (rha) or glucose. Removal of R1 (Rha) results in icariside I, while removal of R2 results in icariside II. Removal of both R1 and R2 results in icaritin. B. Survival curves of N2 hermaphrodites treated with DMSO control or icariin (15, 45 and 75 µM) from day 1 adulthood to death at 25°C. Maximum increase in lifespan was observed at 45 uM. C. Survival curves of N2 treated with DMSO control or icariside II (10, 20 and 40 µM). Maximum increase in lifespan was observed at 20 µM. D. Optimum dosage-response analysis of icariin (45 µM) and icariside II (20 µM). Icariside II treatment induces the similar extension with relatively lower dosage compared to icariin. E. HPLC profiles of icariin and icariside II in N2 treated with 45 µM icariin for 4 days initiated from day 1 in adulthood. HPLC detects high level of icariside II *in vivo*. Small figures (insert) are HPLC profiles of standard samples of icariin and icariside II. All the data above came from 1 representative experiment. For lifespan assay, the treatments were started at day 1 in adulthood continuing to death. Statistical detail and repetitions of the experiments were summarized in Table S1.

We tested icariin for anti-aging properties using wild type *C. elegans* animals. Considering the low bioavailability of icariin, we chose to grow the worms in a liquid medium which has been shown to be more efficient for drug delivery [16]. *C. elegans* N2 worms were exposed to icariin from day 1 of the young-adult stage till their death. Three different concentrations of icariin were used *viz.* 15, 45 and 75 µM. Icariin increases lifespan significantly in a dose-dependent manner (Figure 1B), and among the concentrations examined, 45 µM had the greatest effect on longevity, extending adult mean lifespan by up to 20.67% at 25°C (Figure 1B, Table S1). Worms exposed to either higher or lower concentrations of icariin showed a smaller or an insignificant life extension (Figure 1B, Table S1).

It is well known that the flavonoids obtained from plants are often not the bioactive forms [17]. Pharmacokinetic analysis show that after oral administration, icariin is metabolized to icariside I, by hydrolyzing the C-3-O-Rhamnopyranoside (R1) moiety; to icariside II by hydrolyzing C-7-O-Glucopyranoside (R2) moiety, and to icaritin by the hydrolysis of both the R1 and R2 moieties (Figure 1A). To determine whether the metabolites harbor anti-ageing properties, we tested lifespan effect of these three metabolites in *C. elegans* in parallel with icariin. Neither icariside I nor icaritin showed any significant effect on longevity (Fig. S1A, B; Table S1). However, treatment with icariside II resulted in a lifespan extension similar to that caused by icariin (Figure 1C, Table S1). 20 µM of icariside II treatment is sufficient to cause a lifespan extension similar to 45 µM icariin, suggesting that icariside II might be the bioactive form of icariin *in vivo* (Figure 1D). To investigate this, we measured the internal levels of icariin and its derivatives in wild type *C. elegans* by High-Performance Liquid Chromatography. Worms were treated with the 45 µM icariin for four days starting at day 1 of young–adult stage. The compounds found in worms were mainly icariside II and icariin (Figure 1E). The level of icariside II was higher than icariin (icariin 0.1811 µg; icariside II 0.3154 µg). Icariside I and icaritin were not detected, thus, icariside II is likely to be the primary bioactive structure that possesses anti-aging properties in *C. elegans*. For the remainder of the study, we have used icariside II and the experiments with icariin are presented in the supplemental information.

### Icariside II treatment promotes stress resistance and locomotion in *C. elegans*

**Figure 2:**
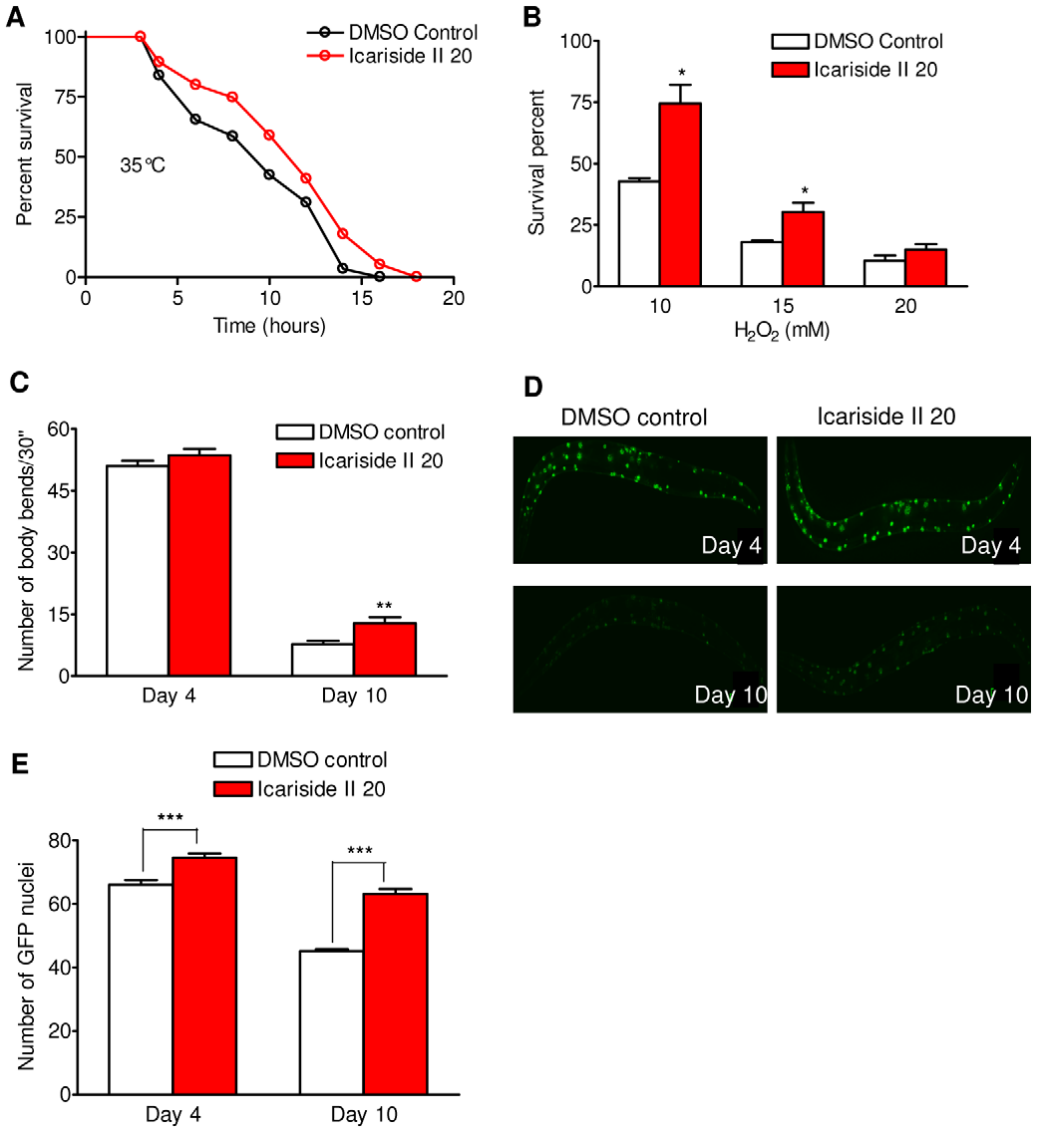
Icariside II promotes stress resistance and slows age related decline in movement in *C. elegans.* A. Icariside II pre-exposure elevated the N2 adults' survival significantly under thermostress. Animals were pre-treated with the DMSO control (1%) or 20 µM icariside II starting from day 1 adulthood for four days. Shown is the combination of two replicates. Mean survival time, DMSO control, 9.7 hrs; icariside II, 11.3 hrs. Animals tested: 87 (DMSO control); 95 (icariside II). *P* = 0.0151 (Log-rank (Mantel-Cox) Test). B. Pre-treatment with icariside II increased oxidative stress resistance significantly. Day 1 wild type adults were treated with DMSO control or 20 µM icariside II for 4 days and then soaked in different concentrations of H_2_O_2_ (10, 15, 20 mM) for 2 hrs. Survival percentages were scored after a 16 hours recovery window in regular NGM. Shown are average survival percentages in 3 experiments with 30–50 animals/experiment. Total number of animals tested: 102 (DMSO Control), 113 (icariside II 20 µM); error bars indicate SEM among three independent experiments; t-test, ^*^ *P*<0.05. C. Treatment with icariside II extends swimming healthspan in advanced age; Average swimming bends per 30 seconds in 15 animals were scored in two independent trials; error bars indicate SEM among individual animals scored; t-test, ^**^ *P*<0.01. D. Icariside II treatment slows the age-related deterioration of *C. elegans* body wall muscle. Age-related deterioration of C. elegans body wall muscle is indicated by GFP fluorescence decline of a p_myo-3_NLS::GFP fusion in the strain PD4251. Representative images of whole-animals treated with DMSO control and icariside II are presented at the ages indicated (25°C). E. Quantitation of the number of fluorescent nuclei in PD4251 treated with DMSO or icariside II. 15 animals of each strain were scored in two independent trials. Error bars indicate SEM among individual animals scored; t-test, ^***^ *P*<0.001.

There is a striking correlation between stresses-resistance and lifespan extension. Compounds that extend lifespan might be associated with improved survival under conditions of heat or oxidative stress. To explore whether icariside II can protect *C. elegans* against vulnerability to stress, we performed the heat stress and oxidative stress resistance assays. In both these assays, N2 hermaphrodites were pre-treated with Dimethyl sulfoxide (DMSO) control or 20 µM icariside II for four days starting at day 1 of young-adult stage at 25°C. Thermotolerance assay was tested at 35°C. Worms were counted every two hours. As shown in Figure 2A, the worms pre-exposed with 20 µM icariside II live significantly longer than the control worms (mean survival time; control, 9.7 hrs; icariside II, 11.3 hrs). A similar protective effect in thermotolerance was also observed upon icariin treatment (Figure S2). In oxidative stress resistance assay, worms were washed at least three times with S basal buffer to exclude the possibility of drug interaction with hydrogen peroxide. Then animals were soaked in hydrogen peroxide (10, 15 or 20 mM) for 2 hrs, transferred to NGM to allow recovery and then scored after 16 hrs. Icariside II pre-treatment afforded dramatic protective effect in the mild (10 mM H_2_O_2_) and moderate (15 mM H_2_O_2_) oxidative stress (Figure 2B; survival percentages; mild, control, 42.8%; icariside II, 74.5%; moderate, control,18%; icariside II, 30.3%).

A major feature of aging is a reduction in muscle strength from sarcopenia, which impairs physical ability and reduces the quality of late life significantly. In *C. elegans*, the swimming bends are a locomotive phenotype which progressively declines with age, indicating physical deterioration of muscle [11]. Wild type N2 were treated with DMSO control or 20 µM icariside II starting from day 1 of young-adult stage. Swimming bends were counted at day 4, which is the beginning of the middle life of N2, and at day 10, which is the start point of the death phase. Our results show that icariside II treated group reduced the decline in swimming prowess at old age (Figure 2C). The number of swimming bends in icariside II treated group is statistically indistinguishable from DMSO control group at day 4 of adulthood. However, the difference in swim vigor is apparent at day 10 (control, 7.7±0.8; icariside II, 12.8±1.4). In order to testify the sarcopenia more directly, we employed a MYO-3::GFP-NLS transgenic strain PD4251. In this strain, the GFP reporter is located in the muscle nuclei, and aging-related deterioration of *C. elegans* body wall muscle is indicated by GFP fluorescence decline [18]. As shown in Figure 2D, GFP labeled nuclei dimmed with aging compared day 4 adults to day 10 adults in control, while 20 µM icariside II treatment reduced the decline of the fluorescence significantly. We quantified GFP nuclei in 15 randomly selected animals in the control and drug treated group at day 4 and day 10. Icariside II treatment was found to delay the decline of quantitation of GFP labeled muscle nuclei significantly (Figure 2E). Together with the swimming assay, these results indicated that both muscle function and integrity are preserved longer by treatment with the compound.

### Icariside II ameliorates protein aggregation and protetoxicity-mediated paralysis phenotype

**Figure 3:**
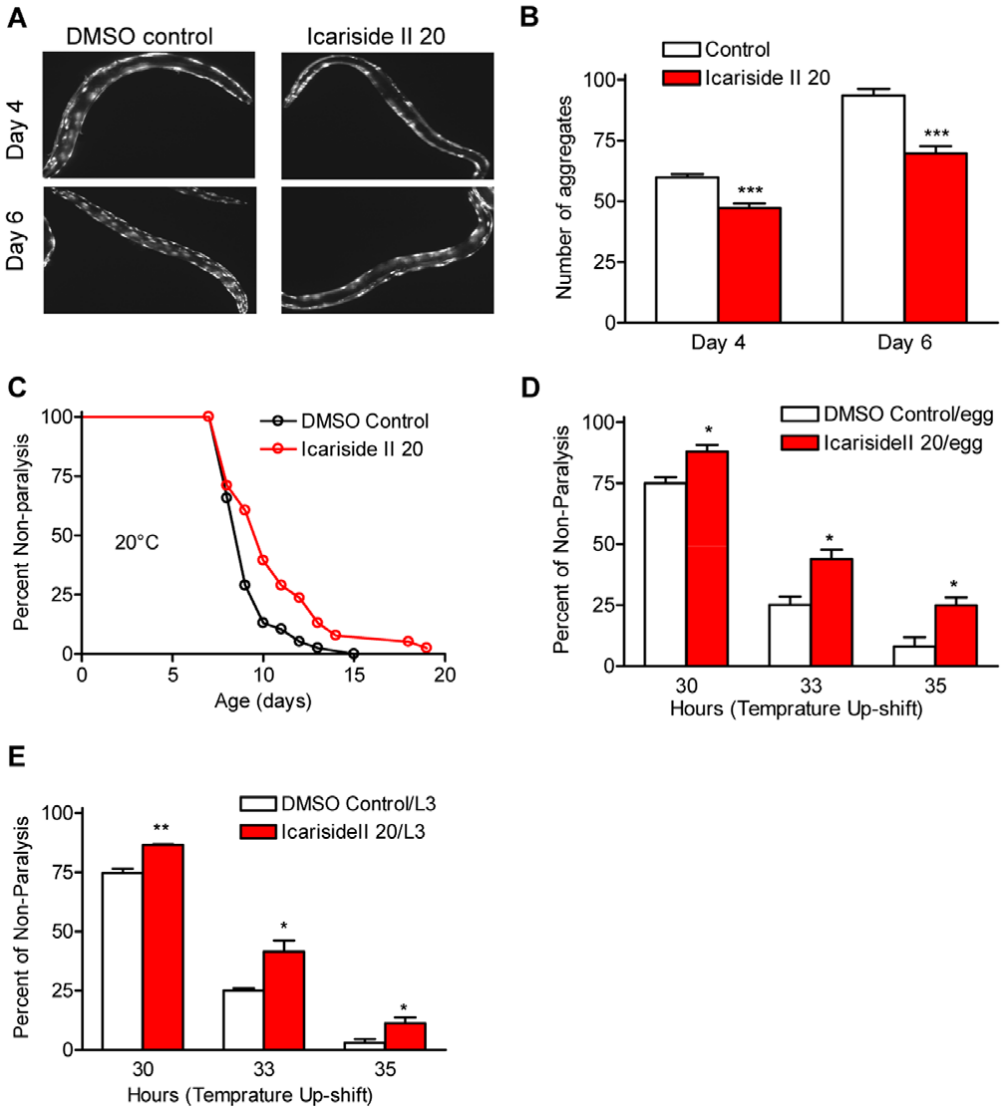
Icariside II ameliorates protein aggregation and protetoxicity-mediated paralysis phenotype. A. Representative images of the whole-animal of Q35-YFP (Q35) transgenic strain AM140 treated with DMSO or icariside II from forth-stage larvae at 20°C are shown. AM140 shows an increasing aggregation phenotype age-dependently. Icariside II treatment slows the aggregation process obviously. B. Quantitation of the number of the fluorescent aggregation in AM140 treated with DMSO or icariside II. 15 animals of each strain were scored in two independent trials. Error bars indicate SEM among individual animals scored; t-test, ^***^ *P*<0.001. C. Age associated paralysis caused by Q35 expression is significantly reduced in icariside II treated animals relative to DMSO control animals. Animal were treated under the conditions from forth-stage larvae until paralysis. Shown is the representative of two replicates. n = 38 (control); 37 (icariside II) animals, *P* = 0.0151 (Log-rank (Mantel-Cox) Test). D. The paralysis phenotype associated with muscle Aβ_1–42_ expression is suppressed by 20 µM icariside II treatment from hatching and E. L3 in the transgenic strain CL4176. Shown is the non-average paralysis percentages in 3 independent experiments with 30–50 animals/experiment in indicated time points after temperature upshift to 25°C; error bars indicates SEM among the non-paralysis percentages of three independent experiments; total number of animals tested: 112 (DMSO Control), 133 (icariside II 20 uM); t-test, ^*^ *P*<0.05, ^**^ *P*<0.01.

Aggregation of misfolded proteins increases with aging and causes a chronic proteotoxic stress which is the main reason for a variety of age-related neurodegenerative diseases, such as Parkinson's disease and Alzheimer's disease [19]. As icariin has a protective effect under conditions of thermal and oxidative stress, we investigated if icariside II can also ameliorate internal proteotoxic stress in *C. elegans*. Two *C. elegans* models of human proteotoxic diseases were exploited here: the strain CL4176 *(dvIs27[pAF29(myo-3/Aβ 1-42/let UTR) + pRF4(rol-6(su1006)])* [20] and AM140 *(rmIs132[P(unc-54) Q35::YFP])* [21]. The strain CL4176 is engineered to provide temperature-inducible muscle expression of a human β-amyloid peptide (Aβ) transgene, resulting in a readable paralysis phenotype of Aβ toxicity upon temperature upshift to 25°C [20]. The strain AM140 contains a YFP fusion with 35 glutamine repeats (Q35-YFP) expressed in the body wall muscle cells which forms aggregates and causes mobility loss as animals age [21]. The accumulation of Aβ is considered to be the central event triggering neuron degeneration in Alzheimer's disease, and polyQ aggregation is a feature in several neurological conditions. We analyzed the age-dependent formation of polyQ aggregation in DMSO control and drug treated animals. Q35-GFP animals were treated with DMSO control or 20 µM icariside II starting from late L4 stage in NGM at 20°C. Fluorescent images were taken at day 4 and day 6 in adulthood. The representative images are presented at Figure 3A as indicated time points. 15 worms were selected randomly for quantification of the aggregates in each group. We found that 20 µM icariside II treatment reduced the formation of polyQ aggregates at day 4 (Figure 3B) and the reduction was higher at day 6 (Figure 3B). The polyQ-dependent paralysis was also significantly postponed in AM140 treated with icariside II (Figure 3C). Same effect was also observed with icariin treatment (Figure S3). Using the Aβ_1–42_ muscle model, we checked whether icariside II protects against Aβ-induced toxicity *in vivo*. Synchronized eggs or L3 larvae were treated with DMSO control or 20 µM icariside II and the treatment was continued for next 60 hrs (for eggs) or 36 hrs (for L3). Aβ induction was induced at L3 stage when the temperature was shift to 25°C. Paralysis at several time points was monitored. A significant delay in paralysis was observed in the transgenic animals with icariside II treatment in both time durations (Figure 3D, egg-duration; Figure 3E, L3-duration), suggesting that icariside II treatment can alleviate the Aβ toxicity and short time treatment (L3-duration) is sufficient to reduce the toxicity.

### Icariside II treatment extends lifespan via IIS pathway

**Figure 4:**
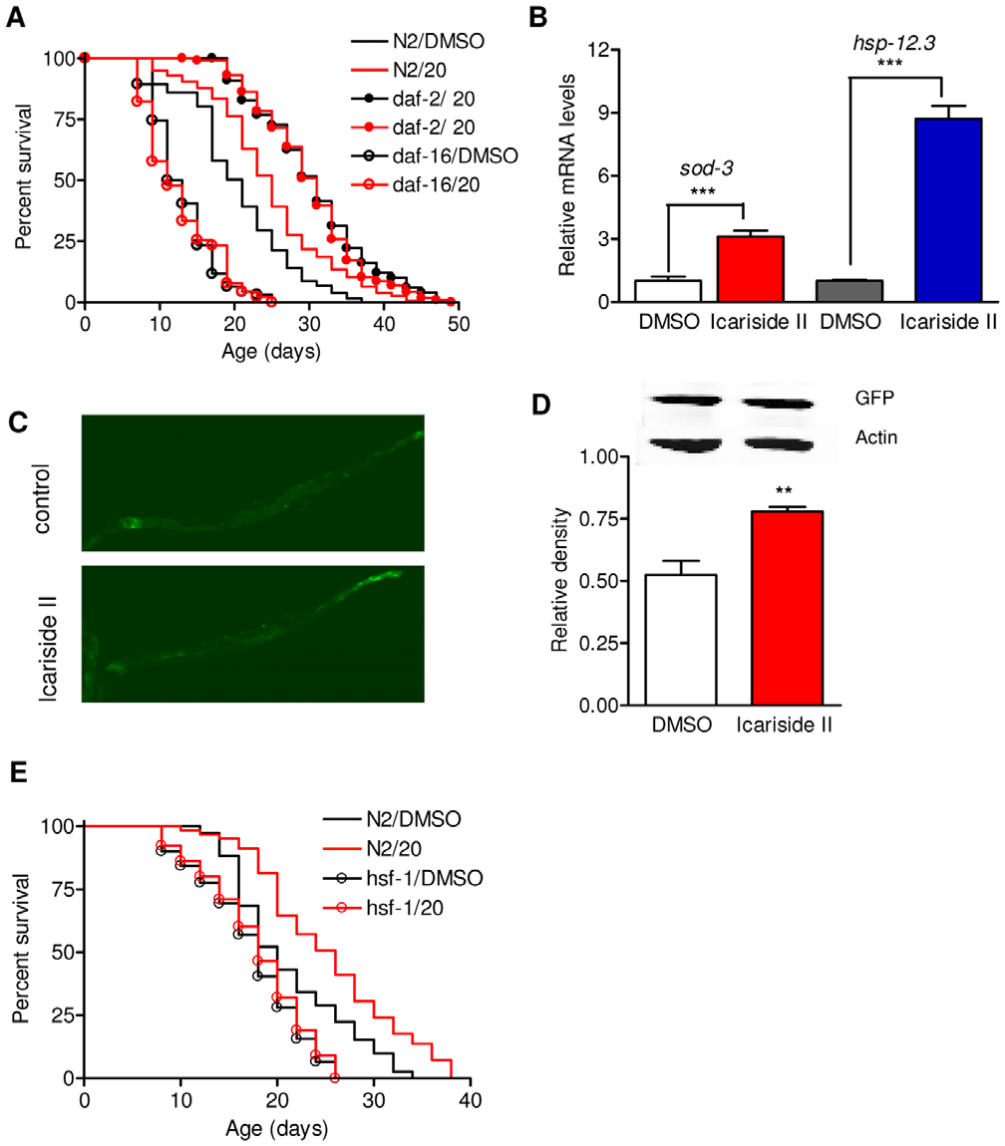
Increased lifespan in *C. elegans* by Icariside II treatment is dependent on the insulin/IGF-1 signaling. A. Survival curves of N2, *daf-2(e1370)*, and *daf-16(mu86)* hermaphrodites treated with DMSO control or 20 µM icariside II are shown. Icariside II treatment does not increase the life span in *daf-2(e1370)* and *daf-16(mu86)* mutant but extends the life span in N2. This is the representative of 3 independent experiments with similar results. Detailed parameters are presented in Table S2. B. Icariside II increases the mRNA expression of FOXO/DAF-16 targets *hsp12.3* and *sod3* significantly. Shown is the representative of three independent experiments with similar results. Error bars indicate SEM among three replicates in qRT-PCR; t-test, ^***^P<0.001. C. Representative images of the whole-animal of SOD-3::GFP transgenic strain CF1553 treated with DMSO or 20 uM icariside II from day 1 in adulthood to day 4 are shown. Icariside II increased the GFP fluorescence significantly compared to DMSO control. D. Western blot analysis shows an increase of GFP induced by 20 uM Icariside II treatment in SOD-3::GFP transgenic strain CF1553. Picture shown is the representative of three independent experiments with similar results. Levels shown are the average relative density level in 3 experiments; error bars indicate SEM among the density level of three experiments; t-test, ^**^ *P*<0.01. E. Effect of icariside II is HSF-1 dependent. Survival curves of *hsf-1 (sy441)* treated with DMSO control and 20 uM icariside II are shown. Icariside II did not increase the lifespan in *hsf-1 (sy441)* mutant.

Down regulation of the insulin signaling pathway increases *C. elegans* lifespan, stress tolerance, locomotory healthspan and resistance to proteotoxicity in a FOXO/DAF-16 dependent way [12]. To investigate whether icariside II increases lifespan by acting through the IIS pathway, the lifespan assay was performed in a *daf-16* null mutant strain. We found that *daf-16(mu86)* mutants raised on 20 µM icariside II did not live longer than the DMSO control group (Figure 4A, Table S2). To gain further insight into the function of icariside II in life span extension by the IIS reduction, we examined the effects of icariside II on the life span in *daf-2(e1370)* mutant. Icariside II fails to lengthen the lifespan in the *daf-2* allele (Figure 4A, Table S2). Together, these results suggest that the life span extensions mediated by icariside II are involved in IIS pathway. The same effect was also observed in icariin treatment (Figure S4).

To ascertain the effect of icariside II on the IIS-DAF-16 pathway, we examined mRNA levels of *sod-3* and *hsp-12.3*, two genes which are activated by DAF-16. SOD-3 is a mitochondrial superoxide dismutase that is involved in oxidative stress resistance and is one of the direct targets of DAF-16 [22]. HSP-12.3 is a small heat shock protein that is activated by DAF-16 at the transcriptional level [22], [23]. We found that *sod-3* and *hsp-12.3* mRNA levels were increased significantly by treatment of 20 µM icariside II for four days starting at day 1 in adulthood (Figure 4B). And in a SOD-3::GFP reporter transgenic CF1553, icariside II treated group showed higher GFP intensity (Figure 4C). Quantitation of the relative intensity in western blot analysis of GFP in CF1553 presented higher GFP level in the icariside II treated group (Figure 4D). These findings provide further evidence that the effects of icariside II acts through IIS pathway.

HSF-1 is a master transcriptional regulator of stress-inducible gene expression and protein folding homeostasis [19]. It acts together with DAF-16 to activate the expression of downstream genes, promoting the longevity in *daf-2* mutant [15]. The HSF-1 is, at least in part, involved in IIS pathway to regulate lifespan, stress resistance and proteotoxicity [24]. We tested the effects of icariside II in *hsf-1(sy441)* mutant. As indicated in Figure 4E and Table S2, the lifespan extension was losted in *hsf-1 (sy441)*, suggesting that the effect of icariside II maybe also HSF-1 dependent.

### Icariside II does not function as DR mimetic

**Figure 5:**
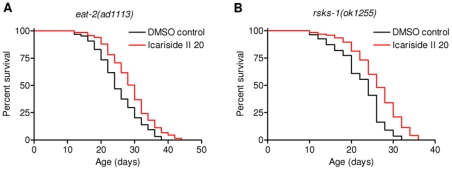
Icariside II may not function as DR mimetic. Survival curves of A. *eat-2 (ad1113)*, B. *rsks-1 (ok1255)* hermaphrodites treated with DMSO control or 20 uM Icariside II is shown. Icariside II treatment caused similar lifespan extension in these mutants as in N2 animals. This is the representative of 2 independent experiments. Detailed parameters are presented in Table S3.

Dietary restriction (DR), the reduction of available nutrients, extends lifespan in diverse organisms from yeast to mammals. It is possible that icariside II may decrease the food intake or interrupt the DR-dependent pathways to extend lifespan. To examine the hypothesis, we performed lifespan assay in *eat-2 (ad1116)* mutant, a well-established genetic DR model [25]. Icariside II treatment caused similar lifespan extension in *eat-2* mutant as in N2 starting at day1 in adulthood (Figure 5A, Table S3). Target of rapamycin (TOR) is an evolutionarily conserved nutrient-sensing kinase which plays a dominant role during DR [26]. Here, our results showed that icariside II extended lifespan in *rsks-1 (ok1255)* mutant (Figure 5B, table S3). *rsks-1* is the homologue of the TOR target ribosomal subunit S6 kinase (S6K) in *C. elegans*. Together, these results indicate that icariside II is unlikely to act as a DR mimetic.

## Discussion

After several decades of efforts, biologists involved in research on aging have produced numerous genes that have been identified to regulate lifespan. Most of them have been associated with a few highly conserved pathways like IIS, TOR pathway, AMP kinase pathway *et al* [12]. Since the discovery of these aging-associated pathways, studies have been performed to uncover pharmaceutical agents that could modify their function. A quantity of anti-aging compounds have been reported: the inhibitor of TOR pathway rapamycin [27], an AMP kinase activator metformin [28], a range of antioxidant agents like vitamin E [29], and a series of antidepressants [30] and anticonvulsants [31]. It is also noteworthy that a group of aging-linked naturally-occurring products belonging to the flavonoid family has been discovered. This group includes compounds like resveratrol, quercetin, catechin, curcumin, a proanthocyanid-rich fraction from blueberry extract and green tea polyphenols [32], [33]. These flavonoids have been shown to extend lifespan and delay the onset of aging-related diseases. And these studies highlight that the flavonoid family as an invaluable resource for the identification of anti-aging compounds. Here, we show that icariin, a flavonol of the herb tonic *Herba epimedii*, along with icariside II, its bioactive form *in vivo*, extends the healthspan in *C. elegans*. We also demonstrate that icariin and icariside II act on the well conserved IIS in aging which strengthens its potential in delaying aging in mammalian system.

In the past, the beneficial effects of flavonoids have mainly been attributed to protection against oxidative stress. However, recent indications point towards a signaling modulator function of flavonoids [17]. Previously studies suggest flavonoids, including icariin, are extensively metabolized *in vivo*, resulting in a significant alteration in their redox potentials [34], [35]. Furthermore, our results show that the C-3-O- Rha moiety is essential for the effect of lifespan extension although the Rha and Glu moieties exert similar redox potentials based on structural analysis [17]. One explanation is that the 3′ position of glycoside may be important for the biological activity of icariin. Many studies show that aglycone form is the main bioactive form of flavonoid [17]. Though icaritin is the aglycone form of icariin, it did not show any effect on lifespan regulation in *C. elegans* in our study (Figure S1B). This reflects the complexity of the action modes of flavonoids as signaling modulators, and the structure-dependence of the effects.

Variations have been existed in the effects of lifespan extension upon flavonoid treatment. For example, Epigallocatechin-3-gallate has been shown to induct a prolongation of *C. elegans* life span in one studies while did not promote longevity in the other two studies [32]. The unstable effects of flavonoid in the longevity tested in *C.elegans* may be caused by several possibilities. One major explanation is the poor absorption and low bioavailability of flavonoids *in vivo* [17]. As indicated by the one of the resveratrol study which shows the lost of lifespan extension without co-solvent utility in *C. elegans* [36], the employment of suitable co-solvent in the culture system is important for elevating the *in vivo* compound level to reach the pharmaceutical activity of the flavonoid. Another notable condition for the compound test in the *C. elegans* system is that liquid culture seems more effective for drug permeability *in vivo* [16]. One possibility is that the compounds are absorbed more efficiently through the penetration of hypodermics of worms in liquid culture. DMSO is an important polar aprotic solvent that dissolves both polar and nonpolar compounds. It is miscible in a wide range of organic solvents as well as water and penetrates the skin very readily. In our study, we use it as co-solvent combined with liquid culture to maximize the penetration of the compounds. In our system, final concentration of 1% DMSO was applied and kept as the control treatment. For the further studies of icariin and icariside II, suitable structure modification can be applied to improve the bioavailability by increase its aqueous solubility.

Increased longevity is not only combating decrepitude but it is also extending the health span, –the period of maintained general health and vigor into late life [11]. Thus we tested a set of parameters which indicate the healthlife upon drug treatment in *C. elegans*. These parameters are constituted by the thermo and oxidative stress resistance, the locomotion and the proteotoxicity. Mutations that extend lifespan in invertebrates typically render the animals resistant to multiple forms of external lethal injury [37]. Locomotion decline is a conserved hallmark of aging. It is thought to be a major underling cause of loss of independence, frailty, and morbidity [11]. The accumulation of structural damage to proteins has been suggested to result in aging, like misfolding, denaturation and aggregation [15], [24], [38]. Here, icariside II enhances the stress tolerance, improving locomotion in late adulthood and ameliorating proteotoxicity in misfolding protein models. Taking together, icariside II treatment leads to a healthspan.

For pharmacological intervention of lifespan, one possibility is that compounds exposure may decrease the pathogenic of bacterial, which have been shown to cause the longevity and good fitness in late life [39]. In our system, this possibility is excluded by adding antibiotics (Carbenicillin, 50 ug/ml) in the culture medium. Another effect for compound test is the hormetic effect, which is depicted as low exposure to stressors enhances the damage repair mechanisms thus increase the lifespan [40]. The possibility that the lifespan extension is the result of a hormetic effect induced by the cytotoxicity of icariside II can also be ruled out, since high doses of icariside II do not cause lethality (Figure S5). Therefore, our data are more supportive for the specific mechanisms of the lifespan extension effects of icariin and icariside II.

Then how could icariside II introduces the beneficial effects in *C. elegans*? These beneficial effects may be the result of an inhibition of a mechanism that normally promotes aging. From *C. elegans* to higher vertebrates, mutations in the insulin-like receptor DAF-2 and its downstream component, PI3K/AKT/PDK kinsases are the essential signaling to enhance longevity and stress tolerance [12]. The FOXO-family transcription factor DAF-16 is considered as the regulatory hub of these phenotypes. Upon a reduction in IIS, DAF-16 is translocated into the nucleus which is released from cytoplasm by the PI3K/AKT/PDK kinase cascade, consorting the expression of the target genes involved in life span control, stress tolerance and protein homeostasis [41]. We proposed that the mechanism of lifespan extension upon icariside II treatment was involved in by IIS restriction in *C. elegans*. Our observations support the proposal. We show that the life span extension is abolished in a null *daf-16* mutant and in a *daf-2* allele suggesting that the mechanisms of icariside II treatment may be overlapped by IIS reduction. Furthermore, we find that icariside II treatment significantly upregulates the mRNA expression of two of the downstream targets of DAF-16 which indicates DAF-16 is activated. Therefore, the IIS pathway is responsible for the mechanisms of icariside II. Heat shock factor 1 (HSF-1) activates transcription of heat-shock genes which encode chaperones and proteases, in response to heat and other forms of stress [15]. Global elevation of heat shock proteins (hsps) is beneficial for the health longevity in IIS restriction background in the invertebrate models [42]. Like DAF-16, HSF-1 is also part of the DAF-2 pathway required for *daf-2* mutants to live long [15]. Our results show that icarisde II treatment does not increase lifespan in *hsf-1* mutant which indicates the HSF-1 is required in the lifespan extension of the compound. Taking together, the results indicate the healthspan extension induced by icariside II via a DAF-2-DAF-16-HSF-1 pathway. As a regulatory center in the lifespan control, several age-associated pathways are directed to act through IIS. It is possible that icariside II may target other pathways then reduces IIS. Recent study shows a novel conserved pathway called EAK (enhancer-of-akt-1) pathway acts parallel to the Akt/PKB kinases to regulate the nuclear DAF-16/FOXO activity [43]. In drosophila, uncoupling protein (UCP) activity specified increased in the insulin-producing neurons reduces IIS signaling and increase lifespan [44]. Post translational modifications like intracellular protein glycosylation is also reported to modulate insulin mediated lifespan in *C.elegans* [45]. Interestingly, Klotho which exerts anti-aging properties in mammals, has been claimed to function through DAF-2/DAF-16 signaling in *C. elegans* [46]. How icariside II restricts IIS to extend lifespan, it is still needed to be investigated in future.

Our findings reveal a novel role for icariin, along with its bioactive form icariside II in extending healthspan via the well conserved pathway, IIS in *C. elegans*. Given the extensive protective effects and safe long term use of icariin and icariside II in humans [2], they may serve as promising anti-aging candidates in the future.

## Methods

### Drug preparation

Icariin, icariside I, icariside II and icaritin were all from Shanghai Winherb Medical Science Co., Ltd with purity (HPLC) 98%, 98.62%, 99.86% and 99.09% separately. All compounds were maintained at 4°C in dark. 100× stock solutions were made with 100% Dimethylsulfoxide (DMSO, Sigma; St. Louis, MO) corresponding to each final concentration in the study. Before adding, 100× stock solutions were diluted to a 10× intermediate solution with S basal freshly.

### Strains

All *C. elegans* strains were maintained at 20°C as described in previously [47], except that temperature-sensitive strains containing AM140, CL4176 and CB1370. The strains used were: N2 Bristol (wild type), PD4251 *dpy-20(e1282) ccIs4251* [P*_myo-3_*NLS/GFP, *dpy-20(+)*], AM140 *(rmIs132[P(unc-54) Q35::YFP])*, CL4176 *(dvIs27[pAF29(myo-3/Aβ 1-42/let UTR) + pRF4(rol-6(su1006)])*, CB1370 *daf-2 (e1370)*, CF1038 daf*-16(mu86)*, CF1553 *muIs84[pAD76(sod-3::GFP)]*, DA1116 *eat-2(ad1116)*, SY3551 *hsf-1(sy441)*, XA8205 *aak-2(ok524)*, XA8223 *rsks-1(ok1255)*.

### Lifespan analysis

Lifespan assay was performed in liquid medium [30] (S-complete medium with 50 mg/ml carbenicillin and 0.1 mg/ml fungizone) in 96-well plates containing, respectively, 150 ml total volume,10–15 nematodes, and 6 mg/ml freshly prepared *E. coli* OP50 per well. Age-synchronized nematodes were seeded as L1 larvae and plates were sealed with tape (Nunc) to prevent evaporation. 5-fluoro-2′-deoxyuridine (0.12 mM final) (Sigma) was added 42–45 h after seeding to prevent self-fertilization. Drugs were added 68 h after seeding (day 1 of adult life) unless otherwise specified. Then the temperature was shifted from 20°C to 25°C. Day 1 of the lifespan assay started 68 h after seeding the animals into plates. The fraction of animals alive per well was scored using a microscope on the basis of movement. Before counting, each plate was put onto a plate rotator for 1–2 min. Strong microscope light (visual) effectively stimulated movement even in old animals. Using this assay, *daf-16*, *daf-2* and *eat-2* mutants showed alterations in lifespan similar to those reported using agar plates.

### Stress resistance assay

For thermotolerance assay, N2 were treated as the method described in lifespan assay, except that the animals were transfer to NGM at day 4 in adulthood. Then animals were put into 35°C and counted every 2 hours. Animals that failed to respond to the prodding against nose were defined as dead. For oxidative stress resistance, N2 were treated as the method described in lifespan assay, except that the animals were washed in M9 buffer 3–4 times then soaked in hydrogen peroxide solution at 10, 15 and 20 mM for 2 hrs at day 4 in adulthood at 20°C. After a 16 hrs recovery period in NGM, survival worms were scored.

### Swimming analysis and GFP-labeled muscle nuclei quantitation

The assays were referred to [11]. Swimming assays were performed at day 4 and day 10 in adulthood. The pre-treatment was the same as the method described in lifespan assay, except that worms in liquid culture were transferred to NGM plates spread with *E. coli* one day ahead the swimming assay. Then individual animals were transferred to 1 mL M9 buffer in a 24-well plate. After a 30-s recovery period, we counted the number of body bends during a 30-s trial using a stereomicroscope for observation. A body bend was defined as a change in the reciprocating motion of bending at the mid-body. Only animals that could move away after a touch and could thrash were used for the swimming assay.

For quantitation of GFP-labeled muscle nuclei of the strain PD4251, the pre-treatment is the same as in lifespan assay. Animals were then paralyzed by 1 mM levamisole, mounted on 1% agarose pads and imaged using Olympus BX51 (60× objective) and HCImage software (Hamamatsu) at day 4 and day 10. For counting the number of GFP-labeled nuclei in the muscle, 15 animals were selected randomly to score the body muscle nuclei. The representative images were given in Figure 2D.

### High Performance Liquid Chromatography (HPLC)

The HPLC system is consisted of a chromatographic pump (Agilent Technologies Series 1200 system, USA)), an autosampler (Agilent, USA) equipped with a 100 µL sample loop, and a DAD detector (Agilent, USA). Icariin, icariside I, icariside II and icaritin were separated using a Waters SunFire™ reversed-phase C18 column (150 mm×4.6 mm, 5 µM) maintained at ambient temperature (25°C). The mobile phase was consisted of acetonitrile and 0.1% aqueous formic acid using gradient elution (0–2 min, 45–80% acetonitrile; 2–5 min, 80% acetonitrile; 5–5.1 min 80–45% acetonitrile) and was delivered at a flow rate of 0.8 ml/min. The mobile phase was filtered through a 0.45 µm Millipore membrane filter prior to use. The DAD detection wavelength was set at 270 nm, and output data from the detector were integrated via a chemstation (Agilent, USA) chromatographic data system. The standard samples of icariin, icariside I, icariside II and icaritin were prepared in methanol at the concentration 10 µg/ml. The current HPLC assay was validated for linearity, intra-day and inter-day precisions, accuracy, extraction recovery and stability. N2 were treated with 45 µM icariin for 4 days as previously stated in lifespan assay. 350–400 worms were collected in M9 with 0.1% sodium azide and washed in deionized water 4–6 times. Then samples were homogenized using a glass stick homogenizator. Samples were sonicated with 4 ml methanol for 10 min and centrifuged at 12,000 rpm for 10 min. The pallet was extracted twice in methanol. The supernatant was dried in a hood overnight to evaporate the methanol. 200 µl methanol was added carefully to dissolve the remaining residue. Then the solution was centrifuged at 12,000 rpm for 10 min. At last 20 µl of the resulting supernatant was filtered using a 0.45 µm syringe filter prior to HPLC analysis.

### Worm paralysis assays and polyQ aggregation quantitation

For strain CL4176 *dvIs2[pCL12(unc-54/humanAb3–42 minigene)1pRF4]*, the method was referred to [20]. Staged populations of CL4176 transgenic worms were prepared by synchronous egg laying and induced to express Aβ as third-stage larvae by upshift from 15°C to 25°C in NGM plates with or without drug treatment. All paralysis plots were done in triplicate with 30–50 worms per condition. Nematodes were scored as paralyzed if they exhibited ‘halos’ of cleared bacteria around their heads (indicative of insufficient body movement to access food) or failed to undergo a full body wave propagation upon prodding. For the strain AM140 *(rmIs132[P(unc-54) Q35::YFP])*, synchronized L4 larvae were transferred to NGM plates with DMSO or drug at 20°C. Worms failed to move forward upon the tail prodding scored as paralyzed. For photograph, animals were paralyzed with 1 mM levamisole mounted on 1% agarose pads and imaged using Olympus BX51 (60× objective) and HCImage software (Hamamatsu) at day 4 and day 10. For polyQ aggregation quantitation, 15 worms from each group were randomly selected to be scored for aggregates at day 4 and day 6. Aggregates were defined as discrete structures with clear boundaries on all sides.

### qRT-PCR assays

N2 animals were treated with DMSO or 20 µM icariside II for 4 days as described in lifespan assay. Then the day 4 adults were collected for total RNA extractions using the Trizol reagent (Invitrogen). The first strand cDNA was synthesized using the reverse transcription system (Qiagen). SYBR Green dye (Quanta) was used for qRT-PCR. Reactions were performed in triplicates on an LC480 machine [48]. Relative-fold changes were calculated using the 2^−ΔΔCt^ method .

### Western blot

For immunoblot analysis, day 4 adults were treated with DMSO control or 20 µM icariside II as described above and replicates of 50 transgenic animals were collected for each treatment in 15 µl HLB buffer (50 mM HEPES-KOH ph7.2; 150 mM NaCl; 1 mM EDTA; 0.1%w/v Sodium deoxycholate; 1%v/v TritonX100; 0.1% SDS) with protease inhibitors . Gel electrophoresis, blotting and detection of specific proteins were performed with standard procedures. Detection and intensity quantitation was performed in an odyssey Infrared Imaging System.

## Supporting Information

Figure S1 **Icariside I and icaritin do not extend lifespan in N2.** A. Survival curves of N2 hermaphrodites treated with DMSO control or 20 µM icariside I from day 1 adulthood to death at 25°C. B. Survival curves of N2 hermaphrodites treated with DMSO control or 20 µM icaritin from day 1 adulthood to death at 25°C. Presented is one of the duplicated experiments. Statistical detail and repetitions of the experiments were summarized in Table S1.
(PDF)

Figure S2 **Icariin enhances thermo tolerance in N2 adults.** Fractional survival percentages of day 4 adults (N2) at the indicated time points at 35°C are increased significantly by icariin treatment. Shown are average survival percentages in 3 experiments with 20–30 animals/experiment. Total number of animals tested: 86 (DMSO Control), 79 (icariside II 20 µM); error bars indicate SEM among three independent experiments; t-test, ^*^ *P*<0.05, ^**^ *P*<0.01.
(PDF)

Figure S3 **Icariin treatment delay polyQ35-mediated paralysis.** Q35-GFP transgenic animals were treated with DMSO control or 45 µM icariin from forth-stage larvae until paralysis. Shown is the representative of two replicates. n = 38 (control); 30 (icariin) animals, *P* = 0.0464 (Log-rank (Mantel-Cox) Test).
(PDF)

Figure S4 **Icariin does not extend lifespan in** ***daf-16*** **and** ***daf-2*** **mutants.** Survival curves of N2, *daf-2(e1370)*, and *daf-16(mu86)* hermaphrodites treated with DMSO control or 45 µM icariin are shown. Icariin treatment does not increase the life span in *daf-2(e1370)* and *daf-16(mu86)* mutant but extends the life span in N2. This is the representative of 2 independent experiments with similar results. Detailed parameters are presented in Table S2.
(PDF)

Figure S5 **High dose of icariside II do not cause lethality in N2.** Survival curves of N2 hermaphrodites treated with DMSO control, 20 and 200 µM icariside II from day 1 adulthood to death at 25°C.
(PDF)

Table S1 **The effects of icariin and its derivates on lifespan in N2.** Mean lifespan of adults in days were observed in lifespan analysis. The different concentrations of compounds tested were indicated. Lifespan assays were performed at 25°C. ‘% change’ was calculated by comparisons to DMSO control of the same experiment. ‘N’ shows the number of observed deaths of animals per experiment. P values were calculated by comparisons to the survival curves of DMSO control of the same experiment using long-rank tests. Individual experiment is listed. ‘*’ indicates the sets of experiments plotted are shown in Figures. Survival curves were plotted and statistical analyses were performed using the Prism 5 software.
(DOC)

Table S2 **Icariside II extends lifespan via insulin/IGF pathway.** Mean lifespan of adults in days were observed in lifespan analysis. The different concentrations of compounds tested were indicated. Lifespan assays were performed at 25°C. ‘% change’ was calculated by comparisons to DMSO control of the same experiment. ‘N’ shows the number of observed deaths of animals per experiment. P values were calculated by comparisons to the survival curves of DMSO control of the same experiment using long-rank tests. Individual experiment is listed. ‘*’ indicates the sets of experiments plotted are shown in Figures. Survival curves were plotted and statistical analyses were performed using the Prism 5 software.
(DOC)

Table S3 **Icariside II does not function as DR mimetic.** Mean lifespan of adults in days were observed in lifespan analysis. The different concentrations of compounds tested were indicated. Lifespan assays were performed at 25°C. ‘% change’ was calculated by comparisons to DMSO control of the same experiment. ‘N’ shows the number of observed deaths of animals per experiment. P values were calculated by comparisons to the survival curves of DMSO control of the same experiment using long-rank tests. Individual experiment is listed. ‘*’ indicates the sets of experiments plotted are shown in Figures. Survival curves were plotted and statistical analyses were performed using the Prism 5 software.
(DOC)
