# Supplementary figures and images for: Icariin and its Derivative Icariside II Extend Healthspan via Insulin/IGF-1 Pathway in C. elegans
Source: PLoS One. 2011 Dec 21;6(12):e28835. doi: 10.1371/journal.pone.0028835 (PMC3244416; doi:10.1371/journal.pone.0028835)

**Figure S1.**

**A**

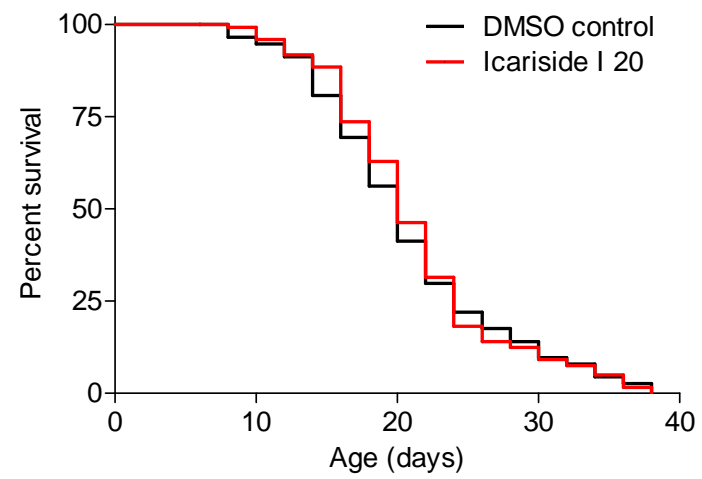

**B**

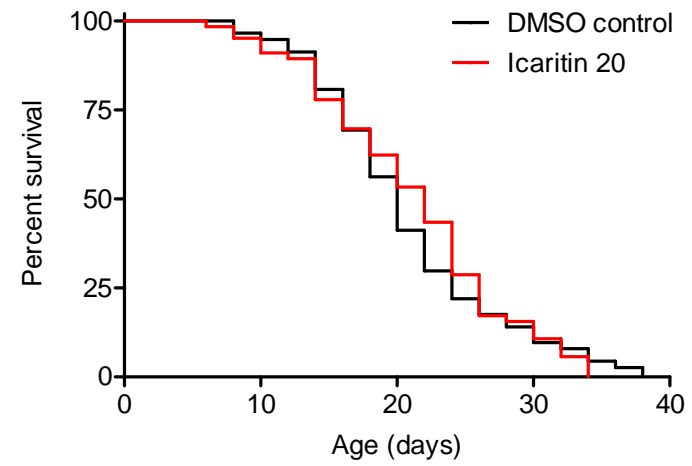

Supplement: Figure S1 — Icariside I and icaritin do not extend lifespan in N2. A. Survival curves of N2 hermaphrodites treated with DMSO control or 20 µM icariside I from day 1 adulthood to death at 25°C. B. Survival curves of N2 hermaphrodites treated with DMSO control or 20 µM icaritin from day 1 adulthood to death at 25°C. Presented is one of the duplicated experiments. Statistical detail and repetitions of the experiments were summarized in Table S1. (PDF) [file pone.0028835.s001.pdf]

**Figure S2.**

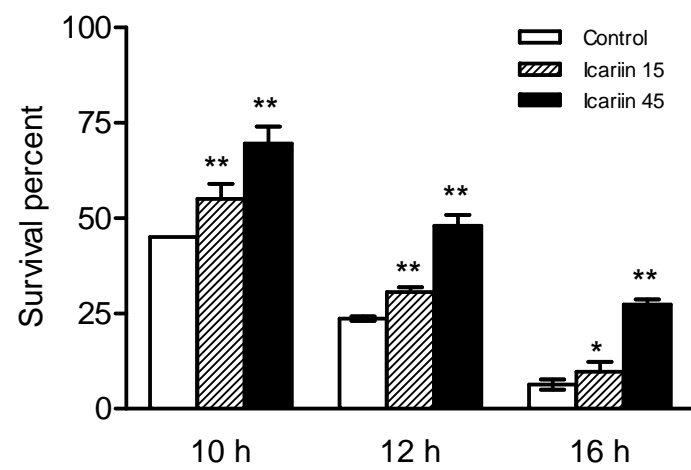

Supplement: Figure S2 — Icariin enhances thermo tolerance in N2 adults. Fractional survival percentages of day 4 adults (N2) at the indicated time points at 35°C are increased significantly by icariin treatment. Shown are average survival percentages in 3 experiments with 20–30 animals/experiment. Total number of animals tested: 86 (DMSO Control), 79 (icariside II 20 µM); error bars indicate SEM among three independent experiments; t-test, * P<0.05, ** P<0.01. (PDF) [file pone.0028835.s002.pdf]

**Figure S3.**

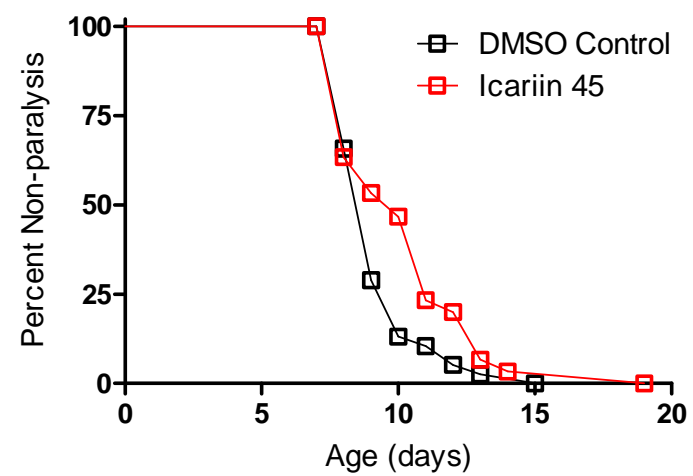

Supplement: Figure S3 — Icariin treatment delay polyQ35-mediated paralysis. Q35-GFP transgenic animals were treated with DMSO control or 45 µM icariin from forth-stage larvae until paralysis. Shown is the representative of two replicates. n = 38 (control); 30 (icariin) animals, P = 0.0464 (Log-rank (Mantel-Cox) Test). (PDF) [file pone.0028835.s003.pdf]

**Figure S4.**

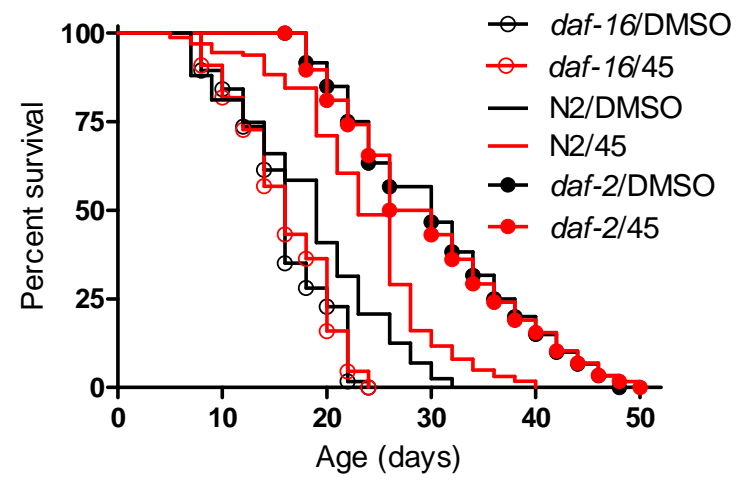

Supplement: Figure S4 — Icariin does not extend lifespan in daf-16 and daf-2 mutants. Survival curves of N2, daf-2(e1370), and daf-16(mu86) hermaphrodites treated with DMSO control or 45 µM icariin are shown. Icariin treatment does not increase the life span in daf-2(e1370) and daf-16(mu86) mutant but extends the life span in N2. This is the representative of 2 independent experiments with similar results. Detailed parameters are presented in Table S2. (PDF) [file pone.0028835.s004.pdf]

**Figure S5.**

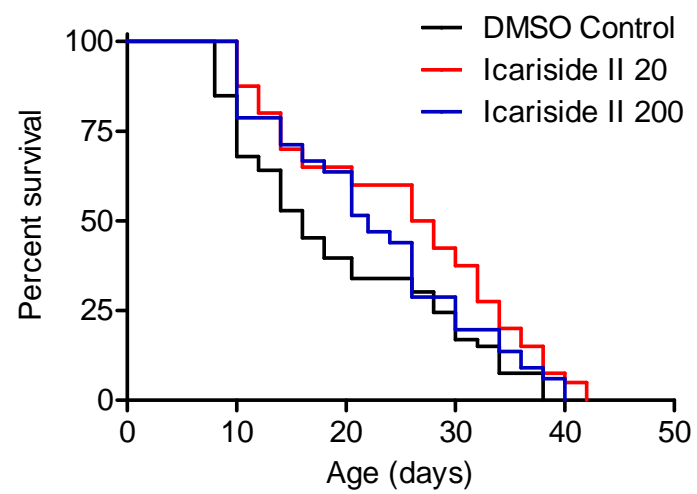

Supplement: Figure S5 — High dose of icariside II do not cause lethality in N2. Survival curves of N2 hermaphrodites treated with DMSO control, 20 and 200 µM icariside II from day 1 adulthood to death at 25°C. (PDF) [file pone.0028835.s005.pdf]
