# Supplementary material for: Icariin and its Derivative Icariside II Extend Healthspan via Insulin/IGF-1 Pathway in C. elegans
Source: PLoS One. 2011 Dec 21;6(12):e28835. doi: 10.1371/journal.pone.0028835 (PMC3244416; doi:10.1371/journal.pone.0028835)
Supplement: Table S1 — The effects of icariin and its derivates on lifespan in N2. Mean lifespan of adults in days were observed in lifespan analysis. The different concentrations of compounds tested were indicated. Lifespan assays were performed at 25°C. ‘% change’ was calculated by comparisons to DMSO control of the same experiment. ‘N’ shows the number of observed deaths of animals per experiment. P values were calculated by comparisons to the survival curves of DMSO control of the same experiment using long-rank tests. Individual experiment is listed. ‘*’ indicates the sets of experiments plotted are shown in Figures. Survival curves were plotted and statistical analyses were performed using the Prism 5 software. (DOC) [file pone.0028835.s006.doc]

| Table S1 Icariin and icariside II extend lifespan dose-dependently | | | | | |
| --- | --- | --- | --- | --- | --- |
|  |  |  |  |  |  |
| Genotypes | Drug treatments (µM) | Mean lifespan (days) | % Change | N | P |
| N2 | DMSO control | 18.3 | -- | 159 | -- |
|  | Icariin 30 | 19.1 | 4.37 | 153 | 0.09 |
|  | Icariin 45 | 23.5 | 28.41 | 162 | < 0.0001 |
|  | Icariin 75 | 18.9 | 3.28 | 172 | 0.7115 |
| N2 | DMSO control * | 20.8 | -- | 114 | -- |
|  | Icariin 30 * | 22.9 | 10.1 | 106 | 0.1787 |
|  | Icariin 45 * | 25.1 | 20.67 | 83 | 0.0054 |
|  | Icariin 75 * | 22.8 | 9.61 | 91 | 0.4158 |
|  | Icariside II 20 | 25.3 | 21.63 | 81 | < 0.0001 |
|  | Icariside I 20 | 21.1 | 1.5 | 121 | 0.7853 |
|  | Icaritin 20 | 21.2 | 1.9 | 122 | 0.6954 |
| N2 | DMSO control | 19.2 | -- | 53 | -- |
|  | Icariin 30 | 21.3 | 10.93 | 42 | 0.0173 |
|  | Icariin 45 | 23.6 | 22.91 | 57 | < 0.0001 |
|  | Icariin 75 | 21.6 | 12.5 | 56 | 0.0821 |
|  | Icariside II 20 | 25.1 | 30.73 | 40 | 0.0133 |
|  | Icariside II 200 | 22.6 | 17.7 | 66 | 0.0764 |
|  | Icariside I 20 | 20.9 | 8.85 | 62 | 0.1876 |
|  | Icaritin 20 | 18.1 | -5.7 | 63 | 0.2656 |
| N2 | DMSO control | 20.7 | -- | 85 | -- |
|  | Icariside II 20 | 25 | 20.77 | 89 | 0.0003 |
| N2 | DMSO control * | 21.3 | -- | 124 | -- |
|  | Icariside II 10 * | 22.4 | 5.2 | 119 | 0.5058 |
|  | Icariside II 20 * | 26 | 22.1 | 126 | < 0.0001 |
|  | Icariside II 40 * | 24 | 12.7 | 127 | 0.0537 |
