# Supplementary material for: Icariin and its Derivative Icariside II Extend Healthspan via Insulin/IGF-1 Pathway in C. elegans
Source: PLoS One. 2011 Dec 21;6(12):e28835. doi: 10.1371/journal.pone.0028835 (PMC3244416; doi:10.1371/journal.pone.0028835)
Supplement: Table S2 — Icariside II extends lifespan via insulin/IGF pathway. Mean lifespan of adults in days were observed in lifespan analysis. The different concentrations of compounds tested were indicated. Lifespan assays were performed at 25°C. ‘% change’ was calculated by comparisons to DMSO control of the same experiment. ‘N’ shows the number of observed deaths of animals per experiment. P values were calculated by comparisons to the survival curves of DMSO control of the same experiment using long-rank tests. Individual experiment is listed. ‘*’ indicates the sets of experiments plotted are shown in Figures. Survival curves were plotted and statistical analyses were performed using the Prism 5 software. (DOC) [file pone.0028835.s007.doc]

| Table S2 Icariside II extends lifespan via insulin/IGF pathway | | | | | |
| --- | --- | --- | --- | --- | --- |
|  |  |  |  |  |  |
| Genotypes | Drug treatments (µM) | Mean lifespan (days) | % Change | N | P |
| N2 | DMSO control * | 20.5 | -- | 207 | -- |
|  | Icariin 45 | 23.8 | 16.1 | 210 | < 0.0001 |
|  | Icariside II 20 * | 24.6 | 20 | 155 | < 0.0001 |
| *daf-16 (mu86)* | DMSO control * | 15.9 | -- | 57 | -- |
|  | Icariin 45 | 16 | 0.62 | 44 | 0.9769 |
|  | Icariside II 20 * | 15.6 | 0 | 48 | 0.6009 |
| *daf-16 (mu86)* | DMSO control | 13.1 | -- | 94 | -- |
|  | Icariin 45 | 13.5 | 3.1 | 75 | 0.3911 |
|  | Icariside II 20 | 12.7 | -3.1 | 90 | 0.8848 |
| *daf-16 (mu86)* | DMSO control | 28.5 | -- | 98 | -- |
|  | Icariside II 20 | 27.3 | -4.2 | 97 | 0.7232 |
| *daf-2 (e1370)* | DMSO control | 30.5 | -- | 60 | -- |
|  | Icariin 45 | 30 | -1.64 | 58 | 0.9514 |
|  | Icariside II 20 | 30.5 | 0 | 61 | 0.705 |
| *daf-2 (e1370)* | DMSO control | 30.3 | -- | 97 | -- |
|  | Icariin 45 | 30.7 | 1.3 | 86 | 0.9737 |
|  | Icariside II 20 | 30.2 | -0.3 | 116 | 0.4579 |
| *daf-2 (e1370)* | DMSO control | 14.4 | -- | 115 | -- |
|  | Icariside II 20 | 14.1 | -2.1 | 120 | 0.2113 |
| *hsf-1 (sy441)* | DMSO control | 15.9 | -- | 96 | -- |
|  | Icariside II 20 | 15.7 | -1.2 | 100 | 0.6802 |
| *hsf-1 (sy441)* | DMSO control | 17.4 | -- | 121 | -- |
|  | Icariside II 20 | 17.9 | 2.9 | 131 | 0.3599 |
