# Supplementary material for: Icariin and its Derivative Icariside II Extend Healthspan via Insulin/IGF-1 Pathway in C. elegans
Source: PLoS One. 2011 Dec 21;6(12):e28835. doi: 10.1371/journal.pone.0028835 (PMC3244416; doi:10.1371/journal.pone.0028835)
Supplement: Table S3 — Icariside II does not function as DR mimetic. Mean lifespan of adults in days were observed in lifespan analysis. The different concentrations of compounds tested were indicated. Lifespan assays were performed at 25°C. ‘% change’ was calculated by comparisons to DMSO control of the same experiment. ‘N’ shows the number of observed deaths of animals per experiment. P values were calculated by comparisons to the survival curves of DMSO control of the same experiment using long-rank tests. Individual experiment is listed. ‘*’ indicates the sets of experiments plotted are shown in Figures. Survival curves were plotted and statistical analyses were performed using the Prism 5 software. (DOC) [file pone.0028835.s008.doc]

| Table S3 Icariside II does not function as DR mimetic | | | | | |
| --- | --- | --- | --- | --- | --- |
|  |  |  |  |  |  |
| Genotypes | Drug treatments (uM) | Mean lifespan (days) | % Change | N | P |
| N2 | DMSO control | 20.7 | -- | 106 | -- |
|  | Icariside II 20 | 24.8 | 19.8 | 118 | < 0.0001 |
| *eat-2 (ad1113)* | DMSO control | 25.2 | -- | 128 | -- |
|  | Icariside II 20 | 28.8 | 14.3 | 133 | < 0.0001 |
| *eat-2 (ad1113)* | DMSO control | 25.1 | -- | 129 | -- |
|  | Icariside II 20 | 29 | 15.5 | 126 | < 0.0001 |
| *rsks-1 (ok1255)* | DMSO control | 22.4 | -- | 110 | -- |
|  | Icariside II 20 | 26.1 | 16.5 | 123 | < 0.0001 |
| *rsks-1 (ok1255)* | DMSO control | 22.2 | -- | 118 | -- |
|  | Icariside II 20 | 26.8 | 20.7 | 142 | < 0.0001 |
